# Clinical implications of a gain-of-function genetic polymorphism in DPYD (rs4294451) in colorectal cancer patients treated with fluoropyrimidines

**DOI:** 10.3389/fphar.2024.1516375

**Published:** 2024-12-05

**Authors:** Elena De Mattia, Jerry Polesel, Lucia Scarabel, Erika Cecchin

**Affiliations:** ^1^ Experimental and Clinical Pharmacology, Centro di Riferimento Oncologico di Aviano (CRO) IRCCS, Aviano, Province of Pordenone, Italy; ^2^ Unit of Cancer Epidemiology, Centro di Riferimento Oncologico di Aviano (CRO) IRCCS, Aviano, Province of Pordenone, Italy

**Keywords:** DPYD, rs4294451, fluoropyrimidine, toxicity, colorectal cancer

## Abstract

Dihydropyrimidine dehydrogenase (DPD, encoded by the *DPYD* gene) is the rate-limiting enzyme for the detoxification of fluoropyrimidines (FLs). Rs4294451 is a regulatory *DPYD* polymorphism that has recently been functionally characterized and associated with increased DPD expression in the liver. The aim of the present study was to test the clinical implications of being a carrier of rs4294451 in a cohort of 645 FL-treated colorectal cancer patients. Carriers of at least one *DPYD* rs4294451-T variant allele had a lower risk of developing NCI-CTC grade 4–5 hematological [odds ratio (OR) = 0.39; 95% confidence interval (CI): 0.15–0.98; additive model] and hematological/non-hematological (OR = 0.44; 95% CI: 0.22–0.88; dominant model) FL-related toxicity. Patients with the *DPYD* rs4294451-T allele also had a longer time to severe toxicity development after starting FL treatment [hematological, Hazard ratio (HR) = 0.27; 95% CI: 0.09–0.79; Fine–Gray test = 0.1569; hematological/non-hematological: HR = 0.38, 95% CI: 0.17–0.85; Fine–Gray test = 0.0444]. It is worth noting that while being at lower risk of toxicity, *DPYD* rs4294451-T allele carriers also tend to present a shorter overall survival (HR = 1.41; 95% CI: 1.05–1.90; log-rank *p* = 0.0406). These findings demonstrate a clinical effect of *DPYD*-rs4294451 polymorphism coherent with the recently described functional effect. Further investigation is warranted to elucidate the potential clinical value to the rs4294451 polymorphism as toxicity and especially as an efficacy marker in colorectal cancer.

## Introduction

Fluoropyrimidines (FLs), mainly 5-fluorouracil (5-FU) and its oral prodrug capecitabine, represent the cornerstone of several antineoplastic regimens currently used to treat a broad spectrum of solid tumors, including gastrointestinal tract, breast, and head and neck cancers (Knikman et al., 2021; Lunenburg et al., 2016). Severe toxicity is observed in up to 30% of patients (Knikman et al., 2021; Lunenburg et al., 2016), with a negative impact on the effectiveness of cancer treatment (i.e., an increased risk of chemotherapy interruption and, consequently, disease progression), patients’ quality of life, and medical costs. For approximately 1% of patients, this toxicity could be fatal (Knikman et al., 2021; Lunenburg et al., 2016). Dihydropyrimidine dehydrogenase (DPD, encoded by the *DPYD* gene) is the first and rate-limiting enzyme in the detoxification pathway of FL, which is constitutively deficient in approximately 3%–5% of Caucasians (Boisdron-Celle et al., 2017). At present, four variants in the *DPYD* gene (DPYD*2A, rs3918290; DPYD*13, rs55886062; c.2846A>T, rs67376798; and c.1236G>A-HapB3, rs56038477) are validated for their clinical impact on FL-related toxicity and recommended for pre-treatment testing by European regulatory agencies according to specific clinical guidelines for drug adjustments (Amstutz et al., 2018; EMA, 2020; Lunenburg et al., 2020). However, the routinely tested four *DPYD* variants identify only a minimal percentage (approximately 17%) of patients experiencing severe FL-related toxicity (De Mattia et al., 2023; De Mattia et al., 2022), and additional validated predictive genetic markers are therefore required. The Association for Molecular Pathology (AMP) PGx Working Group guidelines for *DPYD* testing in clinical practice have recently recommended an extended panel that is more representative of genetic diversity across populations (Pratt et al., 2024).

A recent study (Zhang et al., 2024), using human liver tissues and cellular models to characterize a novel cis-enhancer element capable of modulating DPD expression, has shown that the allelic status of the common germline variant *DPYD* rs4294451A>T could affect CEBPB-driven DPD expression and sensitivity/resistance to 5-FU. Based on the data, the author hypothesized that the higher systemic detoxification of 5-FU due to increased liver DPD expression in carriers of the *DPYD* rs4294451-T allele results in lower exposure to active anti-tumor metabolites of 5-FU. These findings make the *DPYD* rs4294451 polymorphism a strong candidate for the prediction of 5-FU toxicity risk and, potentially, tumor resistance to 5-FU-based therapy. However, the clinical impact of this common genetic variant (22.7% of the minor allele frequency in Non-Finnish Europeans according to gnomAD v3.1.2, https://www.ensembl.org/) has never been tested.

The aim of the present study is to elucidate the clinical role of the *DPYD* rs4294451 variant as predictive pharmacogenetic markers of the clinical outcome (severe toxicity and prognosis) related to an FL-based treatment in a cohort of 645 patients with colorectal cancer (CRC).

## Patients and methods

### Patient cohorts and clinical data collection

Between 1999 and 2019, clinical data and biological samples (blood) from patients receiving FL-based chemotherapy were collected at the Clinical and Experimental Pharmacology Unit of the Centro di Riferimento Oncologico (CRO) in Aviano (PN). From a database of 1,122 clinical cases, the study population was selected based on the following inclusion criteria: (1) diagnosis of colorectal carcinoma; (2) available peripheral biological blood sample; (3) age ≥18 years; (4) assumption of treatment containing FLs (5-FU or capecitabine); (5) available detailed clinical data; and 6) signed written informed. Patients were previously genotyped for DPYD*2A, *13, c2846A>T, and c.1236C>T (Dalle Fratte et al., 2018), and carriers of at least one of the DPYD variants were excluded from the study.

Patients’ medical records were reviewed to collect the following clinical information: (1) baseline clinical–demographic data (e.g., gender, age, and tumor location); (2) chemotherapy information (e.g., FL type, FL starting dose, concurrent administration of chemotherapeutic agents or radiotherapy, start and end dates of therapy, and discontinuation of therapy); (3) toxicity data recorded at each chemotherapy cycle, including severity assessment and start date; (4) patient follow-up data regarding patients’ death.

Adverse events were recorded throughout the entire chemotherapy period and graded according to the National Cancer Institute’s Common Terminology Criteria for Adverse Events v5.0 (NCI-CTCAE). Only toxicities that were more likely to be related to the FL–DPYD interaction were considered, and these were categorized as hematological (i.e., neutropenia) or non-hematological (i.e., nausea, vomiting, diarrhea, hand-foot syndrome, and mucositis). “Any type” toxicity defines both hematological and non-hematological toxicity.

All patients in the study were self-reported Caucasians. The study protocol complied with the ethical guidelines of the 1975 Declaration of Helsinki and was approved by the local ethical committee. All experiments were carried out in accordance with the relevant guidelines and regulations of Centro di Riferimento Oncologico di Aviano (PN).

### Genotyping methods

Patients’ peripheral blood was collected in EDTA-containing tubes. Genomic DNA was extracted from blood samples using the EZ1 DNA Blood 200-mL Kit (QIAGEN) and the BioRobot EZ1 (QIAGEN). Alternatively, genomic DNA was extracted manually using the High Pure Template Preparation Kit. Patients were previously genotyped for DPYD*2A, DPYD*13, DPYD c.2846A>T, and c.1236G>A-*HapB3* (Dalle Fratte et al., 2018).

The analysis of the *DPYD* rs4294451 polymorphism was performed using pre-designed TaqMan SNP Genotyping assays (assay ID: C_32478960_10; functionally tested) according to the manufacturer’s instructions with the TaqMan™ Universal PCR Master Mix on the ABI7500 Real-Time PCR System instrument (Applied Biosystems). Negative and positive controls from previous genotyping were used in each analysis.

### Statistical analysis

#### Socio-demographic and clinical characteristics were reported as absolute frequency and percentage.

To estimate the risk of developing a G4 toxicity associated to the *DPYD* rs4294451 A>T genotype, patients were first classified as having experienced at least an NCI-CTC grade (G) 4 or 5 event (cases) or not (controls). The odds ratio (OR) of developing a G4 toxicity, and the corresponding 95% confidence interval (CI), was estimated through the unconditional logistic regression model, including terms for potential confounders (i.e., gender, age, and cotreatments). Dominant, recessive, and additive genetic models were considered by combining heterozygous with homozygous genotypes; the best-fitting genetic model was selected according to the Wald chi-square test.

Furthermore, to account for the time to G4 toxicity development, a survival analysis was performed. The time at risk of G4 toxicity was calculated from the date of the therapy start to the date of G4 event, death, or last follow-up, whichever occurred first. To account for competing risks, the cumulative incidence of G4 toxicity was calculated (Fine and Gray, 1999), and differences according to the *DPYD*-rs4294451 A>T genotype were tested through Gray’s test (Gray, 1998). Finally, the overall survival was estimated according to the Kaplan–Meier method, and the difference by the rs4294451 A>T genotype was tested through the log-rank test (Kalbfleisch and Prentice, 2002).

## Results

### Patients and genotyping

In total, 689 CRC patients receiving FL-based treatment were selected from our biobank according to the eligibility criteria specified above. Forty-four patients, positive for one of the four previously tested *DPYD* variants, were excluded from the study: DPYD*2A (n = 9), DPYD*13 (n = 0), c2846A>T (n = 8), and c.1236C>T (n = 27). The final study population included 645 cases and the main clinical–demographic characteristics are summarized in Table 1.

**TABLE 1:** Socio-demographic and clinical characteristics of the study population (n = 645).

Characteristic	n	(%)
Gender		
Female	250	(38.8)
Male	395	(61.2)
Age (years)		
<55	145	(22.6)
55–64	210	(32.7)
65–69	131	(20.4)
≥70	157	(24.4)
Tumor localization		
Left colon	173	(26.8)
Right colon	139	(21.6)
Transversal colon	77	(11.9)
Rectum	256	(39.7)
Chemotherapy		
Fluoropyrimidines		
*5-FU*	461	(71.5)
Capecitabine	184	(28.5)
Monotherapy	73	(11.3)
Association with radiotherapy	119	(18.5)
Association with irinotecan	239	(37.1)
Association with oxaliplatin	202	(31.3)
Other	12	(1.9)
Genotype for *DPYD* rs4294451^a^		
AA	438	(67.9)
AT	181	(28.1)
TT	26	(4.0)

^a^ χ2 for Hardy–Weinberg disequilibrium: *p* > 0.05.

*DPYD* rs4294451 (NC_000001.11:g.97930158T>A, GRCh38.p14 chr 1) genotyping was successfully performed for all the 645 patients in the study population. Genotype frequency is given in Table 1.

### *DPYD* rs4294451 variant and toxicity risk

Overall, 60 patients (9.3%) developed at least one G4 toxicity, while no patient experienced G5 toxicity. The distribution of toxicity according to the rs4294451 genotype is given in Table 2. The *DPYD* rs4294451-T allele was significantly associated with reduced risk of developing a G4 hematological (OR = 0.38; 95% CI: 0.14–0.99; additive model) or G4 “any-type” (OR = 0.45; 95% CI: 0.23–0.88; dominant model) toxicity. These associations were confirmed by adjusted logistic regression analysis, both for hematological (OR = 0.39; 95% CI: 0.15–0.98) and “any-type” (OR = 0.44; 95% CI: 0.22–0.88) toxicity. Although not statistically significant, the same trend was observed for the non-hematological toxicity.

**TABLE 2:** Odds ratio (OR) and corresponding confidence intervals (CIs) for G4 toxicity according to the *DPYD* rs4294451 variant. Associations with *p*-value < 0.05 are in bold.

	rs4294451 A>T			Model	OR (95% CI)^a^	OR (95% CI)^b^
	AA (n = 438)	At (n = 181)	TT (n = 26)			
Toxicity type	G4 n (%)	G4 n (%)	G4 n (%)			
Hematological	27 (6.16)	5 (2.76)	0 (0)	Additive	**0.38 (0.14–0.99)**	**0.39 (0.15–0.98)**
Non-hematological	23 (5.25)	5 (2.76)	1 (3.85)	Dominant	0.54 (0.22–1.34)	0.54 (0.21–1.36)
Any type	49 (11.19)	10 (5.52)	1 (3.85)	Dominant	**0.45 (0.23–0.88)**	**0.44 (0.22–0.88)**

^a^ Estimated from the logistic regression model.

^b^ Adjusted for gender, age, and cotreatments (monotherapy vs. multitherapy). “Any type” toxicity includes both hematological and non-hematological toxicity.

**FIGURE 1:**
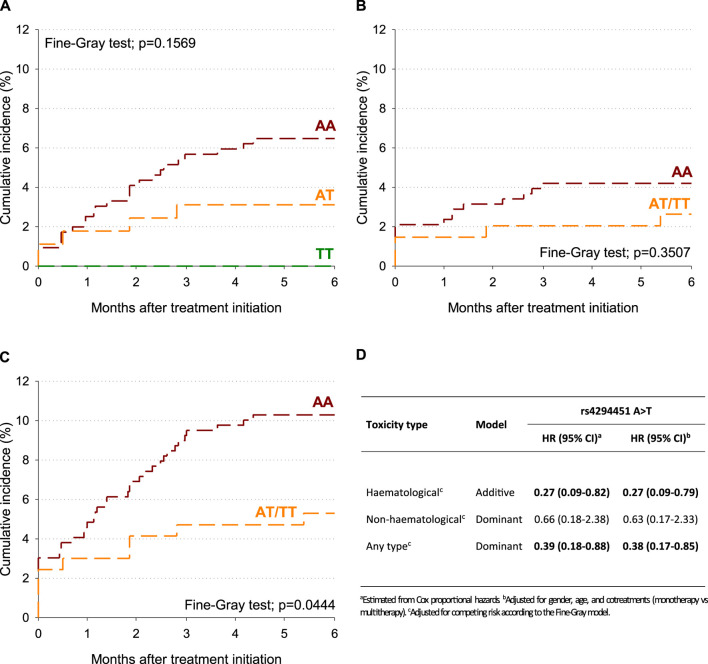
Cumulative incidence of G4 toxicity according to the *DPYD* rs4294451 variant. **(A)** Hematological toxicity. **(B)** Non-hematological toxicity. **(C)** Any-type toxicity. **(D)** Hazard ratio (HR) and corresponding confidence intervals (CIs) for G4 toxicity according to DPYD polymorphisms.

To account for potential competing risk of death, the cumulative incidence of G4 toxicity after treatment initiation was also estimated according to the *DPYD* rs4294451 variant (Figure 1A–C). Within 6 months of treatment initiation, the cumulative incidence of any type of G4 toxicity was 10.3% for the *DPYD* rs4294451-AA genotype and 5.3% for the *DPYD* rs4294451-AT/TT genotypes (Figure 1C; *p* = 0.0444). No significant difference emerged for hematological and non-hematological-specific toxicities (Figure 1A, B), even though the cumulative incidence was lower for *DPYD* rs4294451-AT/TT than for the AA genotype. After adjusting for sex, age, and cotreatments, the rs4294451-T allele was associated with a lower probability of early toxicity development, and this association became statistically significant for the hematological (HR = 0.27; 95% CI: 0.09–0.79; additive model) and “any-type” (HR = 0.38; 95% CI: 0.17–0.85; dominant model) G4 toxicities (Figure 1D).

### *DPYD* rs4294451 variant and overall survival

**FIGURE 2:**
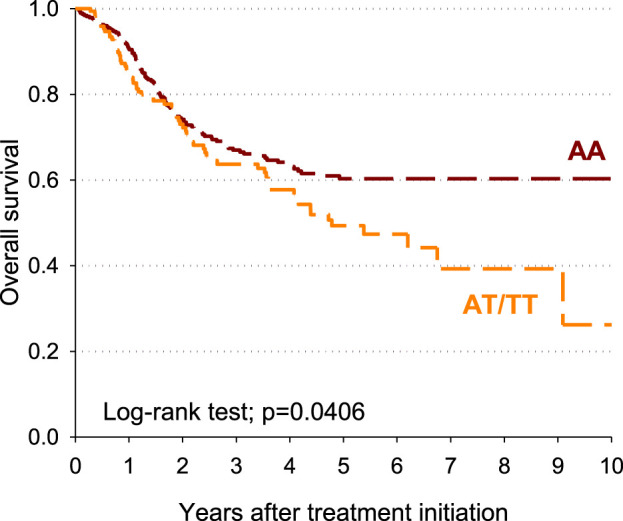
Overall survival according to the rs4294451 variant.

The impact of the *DPYD* rs4294451 genotype on patients’ overall survival was also estimated. Patients carrying the *DPYD* rs4294451 AT/TT genotype had a shorter survival than those with rs4294451-AA, with a 10-year overall survival of 26.2% and 60.3%, respectively (*p* = 0.0406; Figure 2.) After adjusting for sex, age, and cotreatments, a significant excess of risk emerged for the DPYD rs4294451 AT/TT genotype compared to the AA genotype (HR = 1.41; 95% CI: 1.05–1.90).

## Discussion

Despite many years of clinical use and optimization of administration methods, treatment with FL still causes severe toxicity in up to 30% of patients, leading not only to treatment interruption but also patient death in approximately 1% of cases. Pre-treatment *DPYD* genotyping for the four validated markers (DPYD*2A, DPYD*13, c.2846A>T, and c.1236G>A-HapB3) is currently entered into clinical practice and permits the identification of patients at increased risk of developing severe FL-related toxicity before starting therapy (Amstutz et al., 2018; Knikman et al., 2021; Lunenburg et al., 2020). However, the *DPYD* panel of four variants has high specificity (between 99% and 100%) but a low sensitivity (1%–12%) for detecting patients at risk of toxicity (Toffoli et al., 2015), suggesting the need for further investigation of the *DPYD* genotype.

The present study investigated the *DPYD* rs4294451 variant as a potential genetic marker for optimizing FL-based therapy and, for the first time, demonstrated a clinical impact of this polymorphism, which has been recently functionally characterized by Zhang et al. (2024). In particular, in the current analysis, being a carrier of an rs4294451-T allele demonstrated a protective effect against the risk of developing G4 toxicity at any time during treatment and a lower probability of developing early toxicity after starting treatment. At the same time, being a carrier of an rs4294451-T allele was related to a worse prognosis in terms of overall survival. The observed clinical impact was consistent with the functional effect described for *DPYD* rs4294451 polymorphism in the work by Zhang et al. (2024). The rs4294451 polymorphism is located in an enhancer element that controls *DPYD* transcription by recruiting the transcription factor CEBPB (Zhang et al., 2024). Zhang et al. (2024) showed an association between the rs4294451-T allele and increased CEBPB-driven *DPYD* expression, which leads to higher catabolism of 5-FU and lower exposure to the drug. The resulting lower sensitivity to 5-FU associated with the rs4294451-T allele agreed with the decreased risk of developing G4 toxicity reported in the present work for patients carrying the same rs4294451-T allele. These patients also consistently had a poor prognosis in terms of survival.

The present study data support the potential clinical utility of the *DPYD* rs4294451 variant in further refining the genotype-guided FL dosing to prevent severe adverse effects. Previous *DPYD* pharmacogenetic association studies focused on toxicities higher than G3, whereas in the present analysis, we investigated the effect of the variant on extremely high-toxicity events such as life-threatening grade 4–5 toxicities. Those toxicities are of particular clinical importance for both the patient quality of life and management costs, often leading to patient hospitalization and are by far the most relevant to prevent (Le Teuff et al., 2024).

According to gnomAD v3.1.2 (https://www.ensembl.org/), the rs4294451-T allele is quite common, with a frequency of 40.4% in the African/African American population, 22.7% in the non-Finnish European population, and 24.8%–7.3% in the Asian population, making it a good candidate for improving FL treatment optimization based on the patient’s genetic profile. The discovery of novel predictive markers for personalized FL dose adjustment is of particular importance, especially in populations such as African or Asian populations, where the *DPYD* four variants routinely tested in European countries are less common and therefore of less clinical importance.

The *DPYD* rs4294451 has been reported to be in linkage disequilibrium with other two variants located in the *DPYD* coding region, c.85T>C (rs1801265) and c.496G>A (rs2297595) (Zhang et al., 2024). These variants, in haplotype combination with a third variant (c.1129–5923C>G, rs75017182), have been associated with altered systemic DPD activity (Hamzic et al., 2021), which translates into a different risk of severe toxicity (Medwid et al., 2023). Unfortunately, it was not possible in the present study to analyze the clinical impact of the haplotype combination of rs4294451 with other *DPYD* functional polymorphisms, including the four validated *DPYD* markers, because the study sample number was insufficient for this type of analysis. Further studies are warranted to elucidate the effect of an integrated haplotype, including *DPYD* rs4294451 polymorphism, on the clinical outcome of patients treated with FL.

To highlight the specific effect of the *DPYD* rs4294451 variant, patients who were carriers of the four *DPYD* validated markers were excluded from this analysis. The *DPYD* c.1236G>A variant was adopted to tag the HapB3 haplotype. However, it should be noted that a recent report identified rare cases of missing linkage disequilibrium between c.1236G>A and c.1129–5923C>G polymorphism, which is causative for reduced DPD function (Turner et al., 2024).

While most of the studies that investigated the clinical effect of *DPYD* genetics in patients treated with FL focused on the prevention of toxicity, this study reports for the first time a promising impact of rs4294451 on the patient’s prognosis, consistent with the potentially higher detoxification of the drug reported for the polymorphic allele. Since the *DPYD* rs4294451 variant was associated with increased *DPYD* expression, potentially impacting the drug systemic exposure, an open question remains regarding the contribution of this polymorphism to the mechanism of resistance to 5-FU therapy and, consequently, its effect on the clinical tumor response to FL-based therapy. However, due to the heterogeneity of the study population with respect to baseline clinicopathologic characteristics, it is difficult to draw definitive conclusions about the role of the DPYD rs4294451 variant in modulating patient survival. Moreover, the design of the present study was not able to distinguish the predictive or prognostic value of this DPYD polymorphism, so further analyses are needed to better elucidate the impact of the DPYD rs4294451 variant on FL efficacy and survival.

In conclusion, the *DPYD* variant rs4294451 may be a good candidate for further refining the personalization of FL-based therapy based on the patient genotype in terms of both toxicity and efficacy, indicating the need for further research efforts to validate the preliminary data reported here.

## Data Availability

The raw data supporting the conclusion of this article will be made available by the authors, without undue reservation.
